
JOURNAL INFORMATION
==============================
NLM Title Abbreviation: Wirtschaftsdienst
Journal ID: Wirtschaftsdienst
NLM Journal ID: 101086612

Wirtschaftsdienst (Hamburg, Germany : 1949)

ISSN: 0043-6275

ARTICLE INFORMATION
==============================
PMCID: PMC7964464
PMID: 33746307
DOI: 10.1007/s10273-021-2879-4
Article ID: 2879
Article version: 1
Subjects: Ökonomische Trends

Makroökonomische Effekte der Kontaktbeschränkung in der Pandemie

König Michael M.Koenig@fs.de
1
Winkler Adalbert a.winkler@fs.de
2
1 grid.461612.6 0000 0004 0622 3862 FS-UNEP Collaborating Centre for Climate Change u.a., Frankfurt School of Finance & Management, Adickesallee 32-34, 60322 Frankfurt am Main, Deutschland
2 grid.461612.6 0000 0004 0622 3862 International and Development Finance, Frankfurt School of Finance and Management, Adickesallee 32-34, 60322 Frankfurt am Main, Deutschland
Electronic publication date: 2021 Jun 23
Print publication date: 2021
Volume: 101
Issue: 3
First page: 232
Last page: 234
Copyright: © Der/die Autor:in(nen) 2021
License: Open Access: Dieser Artikel wird unter der Creative Commons Namensnennung 4.0 International Lizenz veröffentlicht (https://creativecommons.org/licenses/by/4.0/deed.de).
Open Access wird durch die ZBW — Leibniz-Informationszentrum Wirtschaft gefördert.
License URL: https://creativecommons.org/licenses/by/4.0/

issue-copyright-statement: © The Author(s) 2021

==============================

Ein Jahr nach der ersten Welle ist ein Ende der Corona-Pandemie abzusehen, aber noch nicht in unmittelbarer Reichweite. Weiterhin reagieren viele Regierungen auf das Infektionsgeschehen mit Kontaktbeschränkungen, die sie je nach Fallzahlen entweder verstärken oder reduzieren. Diese auf Verhältnismäßigkeit ausgerichtete Eindämmungsstrategie scheint ökonomisch vernünftig zu sein, da jede Verschärfung bzw. Verlängerung der Maßnahmen hohe Kosten verursacht. Bei fallenden bzw. niedrigen Infektionszahlen sollten Kontaktbeschränkungen und Geschäftsschließungen graduell wieder aufgehoben werden.

Aufgrund der hohen Infektiosität und exponenziellen Entwicklung der Fallzahlen spricht aber auch einiges dafür, schon sehr früh massiv einzugreifen (Hellwig, 2020) und größere Lockerungen erst dann vorzunehmen, wenn die Zahl der Neuinfektionen praktisch Null erreicht hat. Dies ist die Ratio der Eliminierungsstrategie (No-COVID). Aus ökonomischer Sicht scheint sie jedoch mit prohibitiv hohen Kosten einherzugehen, weswegen sie auch als unverhältnismäßig gebrandmarkt wird. Zudem ist sie politisch schwer durchzusetzen, weil der Infektionsverlauf ohne staatliches Eingreifen unbekannt ist: Wären die Infektionszahlen nicht auch ohne einen schnell einsetzenden und lange anhaltenden, harten Lockdown niedrig geblieben, sodass hohe Kosten „für nichts“ produziert werden? Gerade in demokratischen Gesellschaften steht die No-COVID-Strategie daher vor großen Rechtfertigungsschwierigkeiten (Winkler, 2020; Fratzscher, 2021).

Unsere Untersuchung der Zusammenhänge zwischen Infektionsgeschehen, Politikmaßnahmen und ökonomischer Aktivität in OECD-Ländern und einigen aufstrebenden Volkswirtschaften (König und Winkler, 2020, 2021) zeigte bisher, dass in der Tat schärfere staatlich angeordnete Maßnahmen – gemessen am Stringency Index der Universität Oxford (Hale et al., 2020) – das Wirtschaftswachstum über die Zeit stark beeinträchtigten. Allerdings scheint es einen Aufholeffekt zu geben: stärker restriktive Maßnahmen im Vorquartal sind mit einem höheren Wachstum im folgenden Vierteljahr verbunden. Zudem wurde auch deutlich, dass im Ländervergleich eine höhere Zahl der durch und mit COVID-19 ausgelösten Todesfälle (pro 100.000 Einwohner) die wirtschaftliche Aktivität negativ beeinflusste. Insgesamt ließen sich diese Ergebnisse als Bestätigung der Eindämmungsstrategie bewerten, weil sie aus einer rein makroökonomischen Sicht nahelegen, dass Regierungen auf der einen Seite nicht zu strikt vorgehen sollten, auf der anderen Seite aber auch die Pandemie unter Kontrolle behalten müssen.

Im Folgenden wird – soweit Daten zur Verfügung stehen – das vierte Quartal 2020 in die Analyse einbezogen. Zudem wird geprüft, ob Länder, die einer Eliminierungsstrategie gefolgt sind, höhere oder niedrigere Wachstumsverluste im Zusammenhang mit staatlich angeordneten Kontaktbeschränkungen zu beklagen hatten als Länder, die verhältnismäßig vorgegangen sind. Dabei definieren wir Länder mit einer No-COVID-Strategie über die Schnelligkeit mit der Maßnahmen ergriffen wurden, als die Fallzahlen stiegen (Lockdown), und der Zögerlichkeit mit der sie wieder gelockert wurden, als die Fallzahlen sanken (Öffnung). Die Härte des Durchgreifens ermitteln wir anhand der stärksten absoluten Veränderung des von der Oxford University publizierten Stringency Index. Konkret identifizieren wir für das erste und das zweite Halbjahr 2020 den größten 7-Tagessprung des Stringency Index nach oben, also die jeweils stärkste Reaktion auf die erste und zweite Infektionswelle. In Deutschland sind diese größten Sprünge am 22. März (+ 35 Punkte) und am 17. Dezember (+ 15 Punkte) zu registrieren. Daneben greifen wir jenen Tag heraus, an dem die Maßnahmen in der ersten Welle am stärksten gelockert wurden. Dies ist in Deutschland am 5. Mai mit einem Rückgang von 12 Punkten der Fall.

Die Verhältnismäßigkeit dieser Maßnahmen messen wir anhand der dazugehörigen 7-Tagesinzidenz je 100.000 Einwohner. Dabei gilt: je niedriger die Inzidenz desto unverhältnismäßiger ist der Sprung. Denn da sich das Virus exponenziell verbreitet, werden sowohl frühes Handeln bei einem Anstieg als auch späte Lockerungen bei einem Rückgang der Fallzahlen durch eine niedrige Inzidenz angezeigt. Im Falle Deutschlands liegt sie am 22. März bei 23 (Lockdown 1) und am 5. Mai bei 8 (Öffnung 1). Dagegen liegt der Wert am 17. Dezember bei 201 (Lockdown 2). Dies deckt sich mit dem Eindruck, dass in der ersten Welle unverhältnismäßig schnell, in der zweiten Welle dagegen vergleichsweise spät durchgegriffen wurde.

Dennoch: im Vergleich zu anderen Ländern war selbst die Reaktion in der ersten Welle durchaus verhältnismäßig. Dies zeigt die Rangfolge der Summe der 7-Tagesinzidenzen, die an den Tagen der jeweils stärksten Verschärfungen in Wellen 1 und 2 sowie den größten Lockerungsschritten in Welle 1 für das Jahr 2020 gemessen werden (vgl. Abbildung 1). Im Einklang mit entsprechenden Analysen in Wissenschaft (Baker et al., 2020) und Medien (Löhr und Záboji, 2021) platzieren sich China, Australien, Japan, Neuseeland und Südkorea auf den unverhältnismäßigsten ersten Rängen mit einer 7-Tagesinzidenz zwischen praktisch null und sechs. Es folgen Spanien und die Türkei, die aber im Vergleich zu Südkorea einen schon mehr als dreimal so hohen Wert ausweisen.

Wir weisen daher China, Australien, Japan, Neuseeland und Südkorea eine Dummy Variable „Eliminierung“ mit Wert 1 zu, und interagieren diese mit dem Stringenz-Index, sodass der Koeffizient der Interaktionsvariablen darüber informiert, ob und wie sehr die Beziehung zwischen BIP-Wachstum und der Stringenz der vom Staat angeordneten Maßnahmen in diesen Ländern signifikant anders verläuft als in Ländern, die vergleichsweise verhältnismäßig auf das Virus reagiert haben. Wir untersuchen dies in Form einer Panel-Analyse (vgl. Tabelle 1, Spalte 1) für den Zeitraum Quartal 1 2014 bis Quartal 4 2020 sowie OLS-Schätzungen für jedes einzelne Quartal (Querschnittsanalyse, Tabelle 1, Spalten 2 bis 5). Unser Hauptaugenmerk gilt dabei neben dem Interaktionsterm den beiden COVID-Variablen, Stringenz und Todesfälle, sowie der Stringenz für das Vorquartal. Daneben kontrollieren wir für die reale Aktienkursentwicklung, die auch Erwartungen über die Effektivität wirtschaftspolitischer Maßnahmen erfassen soll. In der Querschnittsanalyse kommen weitere, zeitinvariante Kontrollvariablen hinzu. Dazu zählt der Anteil des Tourismus an den Gesamtexporten 2018, der die Anfälligkeit gegenüber im Ausland getroffenen staatlichen und freiwilligen Maßnahmen der sozialen Distanzierung messen soll. Schließlich berücksichtigen wir das BIP pro Kopf 2018 sowie das Trendwachstum der Jahre 2014 bis 2019. Denn es ist davon auszugehen, dass ökonomisch wohlhabendere Länder besser in der Lage gewesen sind, Mittel einzusetzen, um die ökonomischen Folgen der Pandemie abzufedern, während Schwellenländer auch in der Pandemie einen Wachstumsvorsprung gegenüber den Industrienationen aufweisen könnten.

Abbildung 1
**Summe der 7-Tagesinzidenzen bei den größten Veränderungen des Stringency Index, 2020**

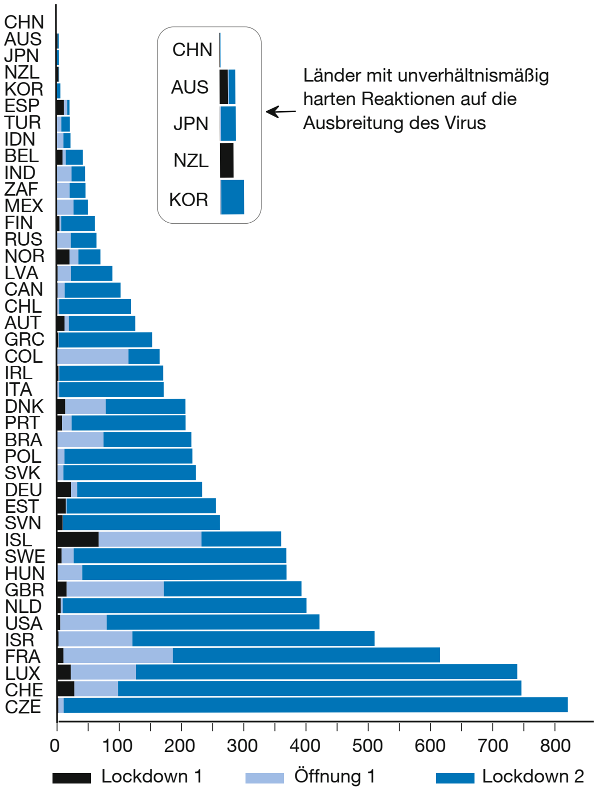

Anmerkung: Länderkürzel nach ISO-3166-1 ALPHA-3.

Quelle: eigene Berechnungen, Oxford University.

Die Ergebnisse bestätigen, dass Veränderungen des Stringenz-Index die Entwicklung des Wirtschaftswachstums über die Zeit hinweg bestimmen (Spalte 1). Dabei lassen sich zwei Effekte unterscheiden: Ein Anstieg des Stringenz-Index um 10 Punkte senkt das Wirtschaftswachstum in diesem Quartal um 1,4 Prozentpunkte (10*(-0,14), vgl. Tabelle 1, Spalte 1, Zeile 1). Allerdings gibt es im Folgequartal einen den Rückgang teilweise kompensierenden Aufholeffekt in Höhe von 0,8 Prozentpunkten. In Ländern, die unverhältnismäßig kräftig auf die Ausbreitung des Virus reagieren, geht ein Anstieg des Stringenz- Index zudem mit niedrigeren Wachstumsverlusten einher: eine Erhöhung um 10 Punkte führt hier „nur“ zu einem Wachstumsrückgang von 0,9 Prozentpunkten (-0,14 + 0,05).

Ähnliche Schlussfolgerungen lassen sich aus zwei der vier OLS Regressionen für die einzelnen Quartale ziehen. Zudem leistet in der Querschnittsanalyse die Zahl der mit COVID-19 in Verbindung zu bringenden Toten einen starken Beitrag zur Erklärung der Wachstumsunterschiede: je höher die Zahl der Toten pro 100.000 Einwohner desto schwächer das Wirtschaftswachstum. Bestätigt wird auch, dass Län-

Tabelle 1
**Wachstumseffekte staatlich angeordneter Kontaktbeschränkungen**

| | (1) | (2) | (3) | (4) | (5) |
|---|---|---|---|---|---|
| Stringenz | -0,14***<br>(0,04) | -0,13*<br>(0,07) | 0,02<br>(0,09) | -0,15***<br>(0,04) | -0,02<br>(0,05) |
| Stringenz*Eliminierung | 0,05**<br>(0,02) | -0,01<br>(0,05) | 0,07*<br>(0,04) | 0,09***<br>(0,02) | 0,04<br>(0,03) |
| Stringenz Vorquartal | 0,08**<br>(0,03) | | 0,07<br>(0,11) | 0,23***<br>(0,06) | 0,02<br>(0,04) |
| Todesfälle (pro 100.000 Einwohner) | -0,02<br>(0,02) | -0,15**<br>(0,07) | -0,11**<br>(0,05) | 0,03<br>(0,05) | -0,04**<br>(0,01) |
| Aktienkursentwicklung | 2,58***<br>(0,82) | -0,54<br>(1,18) | 2,31<br>(2,16) | 2,34<br>(1,49) | 1,21<br>(1,18) |
| Tourismus | | -0,03<br>(0,08) | -0,30***<br>(0,09) | -0,14*<br>(0,07) | -0,19*<br>(0,10) |
| BIP/Kopf (ln) | | -0,07<br>(0,74) | 4,79**<br>(2,12) | 3,36**<br>(1,26) | 0,46<br>(1,22) |
| BIP-Wachstum 2014 bis 2019 | | 0,53**<br>(0,26) | 0,57<br>(0,71) | 1,09***<br>(0,35) | 1,05***<br>(0,35) |
| Konstante | -0,92<br>(0,92) | 2,43<br>(8,93) | -63,75**<br>(26,43) | -51,34***<br>(16,10) | -9,59<br>(13,56) |
| Modell | FE | OLS-Q1 | OLS-Q2 | OLS-Q3 | OLS-Q4 |
| Länder | 42 | 42 | 42 | 42 | 27 |
| $R^2$ | 0,72 | 0,21 | 0,38 | 0,47 | 0,68 |
| F-Wert | 146,32 | 3,74 | 5,36 | 5,60 | 8,52 |

Erläuterungen: Abhängige Variable: BIP Wachstum zum Vorjahresquartal. Alle Modelle mit robusten Standardfehlern. *, **, *** entspricht Irrtumswahrscheinlichkeit von 10 %, 5 %, 1 %, Standardfehler in Klammern. Spalte (1): Panelanalyse mit festen Effekten (Länder, Zeit) beginnend in Quartal 1 2014 bis Quartal 4 2020. COVID-19-Variablen werden bis einschließlich Quartal 4 2019 auf Null gesetzt. In Spalte (2), (3), (4), (5) Querschnittsberechnung für jedes der vier Quartale in 2020, in Quartal 4 mit 27 statt 42 Ländern, da in 15 Ländern, darunter Australien und Neuseeland, noch keine Angaben über das BIP-Wachstum im vierten Quartal vorliegen (König und Winkler, 2021).

Quelle: eigene Berechnungen.

der, die stark vom Tourismus abhängig sind, härter betroffen sind und wohlhabendere Länder sich zumindest in zwei von vier Quartalen besser behaupten können als einkommensschwächere Länder.

Aus diesen Ergebnissen könnte man den Schluss ziehen, dass Deutschland und viele andere Länder eine suboptimale Strategie bei der Bekämpfung von Corona eingeschlagen haben, da die No-COVID-Strategie auch unter ökonomischen Aspekten erfolgversprechend ist. Allerdings ist vor schnellen Schlussfolgerungen zu warnen. Das positive Ergebnis der „Fünf Unverhältnismäßigen" wird maßgeblich von China getrieben. Zudem sind drei dieser Länder Inseln. Hinzu kommt Südkorea als Halbinsel mit der – unabhängig von COVID-19 – wohl undurchlässigsten Landgrenze der Welt. Entsprechend dürften die Abschottungskosten, die eine Eliminierungsstrategie mit sich bringt, deutlich geringer sein als z. B. für Deutschland, Italien oder die USA. Zudem erhöht die Insellage die Glaubwürdigkeit einer No-COVID-Strategie, weil die Wahrscheinlichkeit, auch nach Lockerungen ohne Virus leben zu können, höher ist. Des Weiteren kennt China aufgrund von Erfahrungen mit Epidemien in der jüngeren Vergangenheit die Kosten zu späten Handelns; soziale Kohäsion und politisches System mögen auch dazu beigetragen haben, die Strategie durchzuhalten. Dennoch bleibt die Erkenntnis: Was immer die Besonderheiten dieser Länder sein mögen, sie scheinen aufgrund ihres unverhältnismäßig harten Vorgehens keine ökonomischen Nachteile erlitten zu haben.

Insgesamt ist zu schlussfolgern, dass sich Verhältnismäßigkeit insofern klar auszahlt, als dass jeder Tag mit einem niedrigeren Stringenz-Index – alles andere unverändert – mit geringeren Wachstumseinbußen einhergeht. Wenn jedoch harte Maßnahmen die Zahl der Todesfälle begrenzen, tragen sie in der Querschnittsanalyse indirekt dazu bei, die Wachstumseinbußen zu verringern. Zudem können Länder, die harte Maßnahmen getroffen haben, auf einen Aufholeffekt im Folgequartal setzen. Schließlich weisen Länder, die der Eliminierungsstrategie gefolgt sind, keine stärkeren, sondern eher geringere Wachstumsverluste auf als Länder mit einer Eindämmungsstrategie. Auch wenn daraus keine unmittelbare Empfehlung für die Eliminierungsstrategie abgeleitet werden kann, lässt sich festhalten, dass aus einer rein makroökonomischen Sicht rasches und vermeintlich unverhältnismäßiges Handeln alles andere als nur negativ zu beurteilen ist.

Michael König ist Projekt-Manager am FS-UNEP Collaborating Centre for Climate & Sustainable Energy Finance an der Frankfurt School of Finance & Management in Frankfurt am Main.

Prof. Dr. Adalbert Winkler lehrt International and Development Finance an der Frankfurt School of Finance & Management in Frankfurt am Main.


Literatur

Baker, M. G., N. Wilson und T. Blakely (2020), Elimination could be the optimal response strategy for covid-19 and other emerging pandemic diseases, bmj, 371.10.1136/bmj.m4907 33561814
Fratzscher M Gesellschaftliche Akzeptanz als Schlüssel in der Corona-Pandemie Wirtschaftsdienst 2021 101 2 70 71 10.1007/s10273-021-2838-0 33642640 PMC7896177
Hellwig, M. (2020), Abschied von der heilen Welt, Frankfurter Allgemeine Zeitung, 15. Mai.
Hale, T., N. Angrist, B. Kira, A. Petherick, T. Phillips und S. Webster (2020), Variation in Government Responses to COVID-19, BSG Working Paper Series, BSG-WP-2020/032 Version 6.0, www.bsg.ox.ac.uk/covidtracker.
König M Winkler A Monitoring in real time: Cross-country evidence on the COVID-19 impact on GDP growth in the first half of 2020 COVID Economics 2020 57 132 153
König M Winkler A COVID-19: Lockdowns, Fatality Rates and GDP Growth Intereconomics 2021 56 1 32 39 10.1007/s10272-021-0948-y 33518787 PMC7836344
Löhr, J. und N. Záboji (2021), Zero Covid ist auch keine Lösung, Frankfurter Allgemeine Zeitung, https://www.faz.net/aktuell/wirtschaft/corona-lockdown-die-anhaenger-der-zero-covid-strategie-17164498.html (1. März 2021).
Winkler A COVID-19, Grippewellen und ökonomische Aktivität — die Perspektive der Wirkungsanalyse Wirtschaftsdienst 2020 100 5 344 350 10.1007/s10273-020-2655-x 32454545 PMC7237614
